# Joining Forces Against Antibiotic Resistance in Aquaculture: The Synergism Between Natural Compounds and Antibiotics

**DOI:** 10.3390/antibiotics15010095

**Published:** 2026-01-16

**Authors:** María Melissa Gutiérrez-Pacheco, Martina Hilda Gracia-Valenzuela, Luis Alberto Ortega-Ramirez, Francisco Javier Vázquez-Armenta, Juan Manuel Leyva, Jesús Fernando Ayala-Zavala, Andrés Francisco Chávez-Almanza

**Affiliations:** 1 Departamento de Ingenierías, Tecnológico Nacional de México, Instituto Tecnológico del Valle del Yaqui, Bácum 85276, Sonora, Mexico; 2 Departamento de Ciencias Químico Biológicas, Universidad de Sonora, Hermosillo 83000, Sonora, Mexico; 3 Coordinación de Alimentos de Origen Vegetal, Centro de Investigación en Alimentación y Desarrollo, A.C., Hermosillo 83304, Sonora, Mexico; 4 Departamento de Biotecnología y Ciencias Alimentarias, Instituto Tecnológico de Sonora, Ciudad Obregón 85000, Sonora, Mexico

**Keywords:** antibiotic resistance, virulence, biofilm inhibition, phytochemicals, combination therapy, sustainable aquaculture

## Abstract

The intensification of aquaculture practices has been accompanied by an increased incidence of bacterial diseases, leading to a greater reliance on antibiotics for disease control. Consequently, the widespread and often indiscriminate use of these compounds has contributed to the emergence and dissemination of antibiotic-resistant bacteria within aquaculture systems, posing a serious threat to animal health, environmental sustainability, and public health. In this regard, research efforts have focused on developing alternative strategies to reduce antibiotic use. Natural compounds have gained particular attention due to their well-documented antimicrobial and antibiofilm activities. In this context, the combined application of antibiotics and natural compounds has emerged as a promising approach to enhance antimicrobial efficacy while potentially mitigating the development of resistance. This review synthesizes the current knowledge on antibiotic resistance in aquaculture, highlights the role of biofilm formation as a key resistance mechanism, and critically examines the potential of antibiotic–natural compound combinations against major aquaculture pathogens, with particular emphasis on bacterial growth inhibition, biofilm disruption, and virulence attenuation. Collectively, the evidence discussed underscores the potential of synergistic strategies as a sustainable tool for improving disease management in aquaculture while supporting efforts to limit antibiotic resistance.

## 1. Introduction

Aquaculture has emerged as one of the fastest-growing sectors of food production due to its crucial role in food security and nutrition [1]. Aquatic foods provide nearly 20% of animal protein and are a good source of vitamins, minerals (calcium, zinc), and omega-3 fatty acids, and are consumed at an average of 20.21 kg per capita, per year [2]. The increase in aquaculture production (from 13.1 million mt in 1990 to 82.1 million mt in 2018) has been attributed to the growing population (over 9 billion by 2050) and the increase in seafood consumption [3]. However, the specific nature of aquaculture practices such as the intensification, translocation, and introduction of aquaculture stocks, low genetic diversity, poor water quality, and the high stock densities, makes the organisms particularly susceptible to pathogen virulence and disease development [4]. It has been estimated that up to 50% of aquaculture production is lost due to diseases [5]. Among the most common bacterial pathogens affecting aquaculture settings are some belonging to the genera *Aeromonas*, *Vibrio*, *Pseudomonas*, *Streptococcus*, and *Edwardsiella* [6].

In this sense, antibiotics have been extensively used for the treatment of diseases and, in some cases, for prophylactic purposes. However, their widespread and often indiscriminate use has contributed to the emergence and spread of antibiotic-resistant bacteria in aquatic environments, causing severe impacts on animal health, environmental sustainability, and public health [7]. In fact, this phenomenon is exacerbated by antibiotic residues that persist in water, sediments, and the tissues of cultured organisms, creating a selective pressure that promotes the emergence and maintenance of antibiotic-resistant bacteria and resistance genes [8].

Among the mechanisms contributing to antimicrobial resistance, biofilm formation has gained particular attention. Biofilms are microbial communities in which microorganisms are enclosed in an extracellular polymeric matrix that protects bacteria from antibiotics, disinfectants, and host immune responses [9]. In aquaculture settings, biofilms can readily develop on biotic and abiotic surfaces, leading to persistent infections and recurrent disease outbreaks [10]. Bacteria embedded within biofilms can exhibit tolerance levels even up to 1000 times compared with their planktonic counterparts, rendering conventional antibiotic treatments largely ineffective [11]. This limitation highlights the need for strategies that not only inhibit bacterial growth but also target biofilm formation and virulence regulation.

In this context, natural compounds are promising alternatives and adjuvants to conventional antibiotics. These compounds have demonstrated antibacterial activity against aquaculture pathogens [12]. Beyond direct growth inhibition, many natural compounds have been shown to disrupt biofilm architecture, interfere with quorum sensing and other regulatory pathways, and attenuate bacterial virulence. These multifaceted effects highlight their potential for use in combination therapies, potentiating the antibiotic activity, reducing the effective doses required to achieve bacterial control, and antibiotic resistance. Despite the growing number of studies reporting the synergistic antibacterial and antibiofilm effects, most evidence is still limited and poorly integrated, mainly based on in vitro assays. Moreover, the applicability of these findings under real aquaculture conditions has been insufficiently addressed. Challenges such as formulation stability in aquatic environments (degradation, loss of effectiveness), cost-effectiveness at farm scale, delivery routes, potential impacts on non-target microbiota or in organism’s physiology, and regulatory approval need to be considered and evaluated. Addressing these gaps is essential to determine whether such combinations can provide consistent, safe, and effective outcomes in practical production settings. This review aims to critically analyze these limitations and highlight how synergistic antibiotic-natural compound combination strategy may enhance antibacterial efficacy, disrupt biofilms, and mitigate resistance in major aquaculture pathogens.

## 2. Aquaculture Bacterial Diseases

Aquaculture has become one of the fastest-growing sectors of food production worldwide, providing healthy and nutritious aquatic foods to the population and significantly contributing to food security [13]. It has been estimated that about half of the fish and seafood consumed worldwide is produced by aquaculture. According to The State of World Fisheries and Aquaculture [14], in 2022, global aquaculture output exceeded capture fisheries for the first time, reaching 130.9 million tones and accounting for 51% of total aquatic animal production. However, the rapid expansion of aquaculture has been accompanied by a higher incidence of infectious diseases, which are now one of the major threats to its sustainability. This increased disease burden is strongly influenced by fluctuations in temperature, oxygen, salinity, pH, and organic and inorganic nutrients, which favor pathogen proliferation [15]. The Food and Agriculture Organization (FAO) reported that the most important bacterial diseases in aquaculture include Vibriosis, Aeromoniasis, Edwardsiellosis, Pseudomoniasis, Flavobacteriosis, Mycobacteriosis, Streptococcosis, Renibacteriosis, and infections caused by intracellular and anaerobic bacteria. Each of these diseases affects different aquaculture species and is associated with a wide range of clinical signs (Table 1) [6]. The severity and manifestation of symptoms vary depending on the bacterial agent involved, the host species, environmental conditions, and the stage of development of the cultured organisms.

*Vibrio* species are widely distributed in aquatic environments and are among the most common pathogens affecting aquaculture systems. Several species are recognized as foodborne pathogens, posing a risk to human health through the consumption of contaminated seafood [16]. At the same time, other species are responsible for severe diseases in farmed aquatic organisms, such as shrimp and fish, resulting in high mortality rates and substantial economic losses in aquaculture production worldwide [17]. Among these, *V. parahaemolyticus* is one of the most important pathogens in aquaculture. This microorganism can be associated with organic matter, plankton, and aquatic organisms. Among their hosts, the white shrimp (*P. vannamei*) is the most affected [18]. There are pathogenic strains of *V. parahaemolyticus* that harbor a pVA1-like plasmid carrying the genes *pirA* and *pirB* encoding the toxins responsible for one of the most devastating diseases in shrimp farming, the Acute Hepatopancreatic Necrosis Syndrome (AHPND), also known as Early Mortality Syndrome (EMS) [19]. The AHPND-causing strains can lead to mortality rates of up to 100% in the early culture stages, mainly due to the massive hepatopancreatic damage [15].

Another important pathogen in aquaculture is *A. hydrophila* [20]; this bacterium is commonly found in aquatic environments such as estuaries, sediment, and seaweed, as well as in foods and food-processing facilities [20,21]. Changes in environmental factors, including temperature, salinity, pH, dissolved oxygen, overfeeding, and malnutrition, among others, caused stress to cultured organisms, making them more susceptible to *A. hydrophila* infections [22]. *Aeromonas* spp. cause diseases such as hemorrhagic septicemia and ulcerative syndrome in some fishes (Table 1), which cause high mortality rates and economic losses in aquaculture. The pathogenicity of *A. hydrophila* is associated with the synthesis and/or secretion of virulence factors that enable it to colonize host tissues, damage cells, and evade host defenses in fish. Examples include hemolysins, cytotoxins, adhesins, and extracellular enzymes; however, their relevance extends beyond host damage to mechanisms that promote persistence such as the ability to form biofilms [22,23]. A direct association between *A. hydrophila* biofilms and antibiotic resistance was demonstrated in the study of Abo-Shama, El Raheem [24] it was reported that the 100% of *Aeromonas* isolates from infected *O. niloticus* were biofilm producers and 79.5% were multidrug-resistant.

Streptococcosis caused by *S. agalactiae* is an economically important bacterial disease that results in significant losses to the aquaculture industry. In this sector, *S. agalactiae* is the primary cause of streptococcosis in tilapia (*Oreochromis* spp.) and causes significant mortality worldwide [25]. The disease causes several symptoms (Table 1) and high mortality rates. The capacity of this bacterium to cause disease is attributed to the presence of a capsular polysaccharide that protects the bacteria from phagocytosis, thereby enabling the pathogen to survive. The synthesis of β-hemolysin/cytolysin contributes to the development of necrosis and hemorrhage due to its capacity to destroy erythrocytes, epithelial cells, and leukocytes. In addition, the fibrinogen-binding proteins (FbsA and FbsB) and the fibronectin-binding protein (PavA) are key factors in adhesion to epithelial and endothelial cells, contributing to bacterial colonization and invasion, developing biofilms on the fish gills and intestine [26].

*E. tarda* is the primary causal agent of Edwardselliosis, the most common disease affecting catfish farms [27] (Table 1). The infection can occur in both intensive and extensive systems, most frequently when the temperature is high, oxygenation is low, or there is an excess of organic matter, as these conditions promote bacterial proliferation [28]. One important virulence factor of *E. tarda* is hemolysins, which cause septicemia by destroying the membranes of erythrocytes, leukocytes, and endothelial cells, leading to cell lysis, promoting bacterial multiplication and septicemia. Additionally, it has been reported that virulence genes such as *qseC* are present in some pathogenic *E. tarda* strains [29]. *qseC* is a histidine kinase sensor that participates in the quorum-sensing (QS) system, which regulates several phenotypes, including genes related to biofilm formation [30].

In particular, the pathogenicity of each bacteria is linked to its specific virulence factors, such as toxins, adhesion molecules, secretion systems, immune evasion mechanisms, and biofilm formation [31,32]. These factors determine the bacteria’s ability to invade, colonize, and damage host tissues, thereby influencing the clinical outcome of the infection. Overall, bacterial diseases in aquaculture are closely linked to pathogen virulence and environmental stress, and their control has primarily relied on antibiotic use. This practice has accelerated the emergence of antibiotic-resistant bacteria, making antimicrobial resistance a critical issue for the sustainability of aquaculture.

## 3. Antibiotic Resistance in Aquaculture

### 3.1. Understanding Antibiotics in Aquaculture: Impact and Usage

An antibiotic is an antimicrobial substance derived from natural sources, such as bacteria or fungi, or synthesized in laboratories. Antibiotics specifically target and kill or inhibit bacterial growth, making them crucial for treating bacterial infections in humans, animals, and plants [33]. They work by attacking various structures or functions of bacterial cells, such as cell wall synthesis [34], protein production [35], DNA replication [36], or metabolic pathways, without harming the host’s cells. This selective toxicity makes antibiotics powerful tools for managing bacterial diseases in many fields, such as medicine, agriculture, and aquaculture. Antibiotics can be classified according to (1) chemical structure, as β-lactams, tetracyclines, aminoglycosides, macrolides, quinolones/fluoroquinolones, sulfonamides and glycopeptides [37,38,39,40,41,42,43]. They also can be classified according to their (2) mechanism of action into cell wall, proteins, or nucleic acid synthesis inhibitors [34,35,36]. Other classifications are based on their spectrum activity (broad- or narrow-spectrum) and origin (natural or synthetic) (Table 2).

The use of antibiotics may have existed before their formal discovery, but the antibiotic era is generally considered to have begun with Sir Alexander Fleming’s discovery of penicillin in 1929. Antibiotics have since been crucial in modern medicine, saving countless lives and increasing life expectancy [44]. Soon after their introduction in human medicine, new antibiotic compounds were also made available for use in aquaculture. Their use in aquaculture began with the intensification of farming practices, as maintaining healthy populations in often stressful conditions became essential. Antibiotics are commonly used in aquaculture mainly to treat bacterial diseases and, to a lesser extent, to prevent them. According to a survey by Chowdhury, Rheman [45], in Bangladesh, 71% of surveyed farms report antibiotic usage at least once since the start of their production cycle. Despite the widespread use, only 9% of farmers administer antibiotics prophylactically on the first day, with most relying on antibiotics only when disease symptoms appear. Approximately, 73% of major aquaculture-producing countries (around 73%) reported using oxytetracycline, florfenicol, and sulphadiazine; whereas 55% reported the use of erythromycin, amoxicillin, sulfadimethoxine, and enrofloxacin [46].

**Table 2:** Commonly used antibiotics in aquaculture based on their chemical structure.

Class of Antibiotic	Antibiotics	Structure	Mode of Action	References
β-lactams	Penicillin G	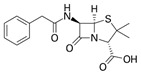	Inhibition of cell wall synthesis	Kim, Kim [47]
	Amoxicillin	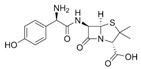		
	Cephalexin	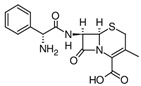		
Tetracyclines	Tetracycline	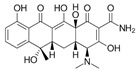	Inhibition of protein synthesis	Guidi, Santos [48]
	Oxytetracycline	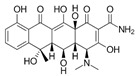		
	Doxycycline	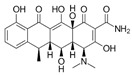		
Fenicols	Florfenicol	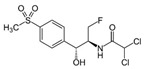	Inhibition of protein synthesis	Trif, Cerbu [49]
	Thiamphenicol	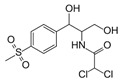		
Aminoglycosides	Gentamicin	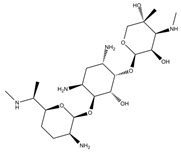	Inhibition of protein synthesis	Shi, Caldwell [35]
	Streptomycin	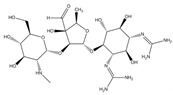		
	Neomycin	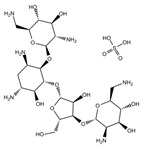		
Macrolides	Erythromycin	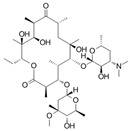	Inhibition of protein synthesis	Vázquez-Laslop and Mankin [40]
	Azithromycin	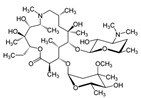		
	Clarithromycin	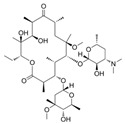		
Quinolones/fluoroquinolones	Enrofloxacin	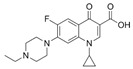	Inhibition of DNA replication and transcription	Sousa, Alves [36]
	Ciprofloxacin	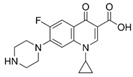		
	Norfloxacin	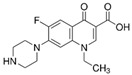		
Sulfonamides	Sulfamethoxazole	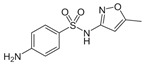	Inhibition of bacterial growth	Ovung and Bhattacharyya [42]
	Sulfamethazine	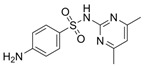		
	Sulfadimethoxine	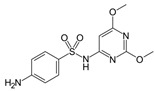		

Several factors, including culture methods and species susceptibility, influence antibiotic use in aquaculture and determine the resulting concentrations of these compounds in water, sediments, and farmed organisms. For example, enrofloxacin has been found in Mozambican tilapia (*Oreochromis mossambicus*), Nile tilapia (*O. niloticus*), climbing perch (*Anabas testudineus*), giant snakehead fish (*Channa micropeltes*), etc., in doses of 355 µg/kg, being its maximum residue limits (MRL) of 200 µg/kg [50]. Whereas norfloxacin in 3.1–100.5 µg/kg on farmed freshwater fish (MRL = 100 µg/kg) [51]. Similarly, oxytetracycline, the most used antibiotic, was found in *O. niloticus* at 10–1319 µg/kg [52]. On the other hand, 15,090 µg/kg of erythromycin was detected in the redtail shrimp *Fenneropenaeus penicillatus* [53].

Importantly, antibiotic residues detected in aquatic organisms and surrounding environments frequently reflect subinhibitory exposure levels rather than bactericidal concentrations. Several studies have reported that the elimination half-life of antibiotics in aquatic organisms varies considerably depending on both the species and the compound used. For instance, in shrimp (*P. vannamei*), oxytetracycline exhibits an elimination half-life of approximately 22 h, indicating moderate persistence in tissues [54]. In the same species, florfenicol shows a much shorter plasma half-life (≈0.35 h); however, residues remain detectable for up to 24 h [55]. This highlights that a short plasma half-life does not necessarily imply rapid or complete elimination of the antibiotic from the organism. In farmed fish such as rainbow trout (*O. mykiss*), florfenicol displays prolonged elimination half-lives in plasma and tissues (≈20–28 h), demonstrating greater antibiotic retention compared with crustaceans [56]. Under aquaculture conditions, factors such as dilution in water, adsorption to sediments, and uneven feed consumption further reduce effective drug bioavailability. These conditions do not eliminate pathogenic bacteria but instead favor adaptive responses, including stress tolerance, resistance selection, and enhanced biofilm formation [57].

Additionally, Han, Zhang [31] showed that the distribution of antibiotics across culture systems (outdoor ponds, greenhouse ponds, and shallow sea cultures) differed, with the greenhouse culture system showing the highest antibiotic concentrations. The observations were attributed to prolonged culture duration, high stocking densities, poor air circulation, limited light, and restricted water exchange. Also, the shift to more intensive practices in shrimp farming led to increased disease outbreaks and, consequently, higher antibiotic use [58]. On the other hand, species with weaker or less adaptive immune systems, like bivalves and snails, often require more antibiotics to combat bacterial infections [59]. Conversely, in salmon farming, shifting from Pacific to Atlantic salmon, which are less susceptible to certain diseases, significantly reduced antibiotic use due to better disease management and available vaccines [60]. These findings underscore the need for tailored antibiotic management strategies in aquaculture that account for both specific culture methods and the biological characteristics of farmed species to minimize environmental impact and promote sustainable practices.

Several issues arise from the improper use of antibiotics associated with limited knowledge, indiscriminate use, unawareness of residual effects and expiration dates, and inadequate diagnostic facilities for accurate disease identification [61,62]. These practices lead to the overuse and misuse of antibiotics in aquaculture, which can accumulate in sediments, water bodies, and animals, promoting the growth of antibiotic-resistant bacteria. The presence of antibiotics in coastal and surface waters has become an escalating environmental concern. Studies, such as those conducted in Dalian’s coastal waters of the Bohai Sea by Du, Zhao [63], have highlighted the widespread detection of antibiotics with total concentrations ranging from 22.6 to 2402.4 ng/L. Antibiotics such as enrofloxacin are frequently detected in these waters and are sufficient to exert selective pressure on bacteria, thereby promoting the development of antibiotic resistance [63]. Similarly, a study in the East China Sea conducted by Minich, Poore [64] indicated that multiple antibiotics, including clindamycin and erythromycin, were prevalent in coastal water and sediment. The joint toxicity of these antibiotics was found to be more severe than the individual effects, particularly nearshore, where concentrations were higher.

On the other hand, antibiotics in commercialized marine products are frequently reported. Antibiotics in seafood, such as fish and shrimp, pose a significant risk to food safety and public health. A study conducted in Saudi Arabia found that antibiotics like oxytetracycline, tetracycline, and chlortetracycline were present in 24% of seafood samples, with 15% exceeding the MRL. Notably, canned fish had the highest levels of oxytetracycline, with over half of the samples surpassing the MRL [65]. Other studies have shown a link between seafood antibiotic residues and the development of antibiotic resistance. In Brazil, a study on tilapia (*O. niloticus*) fillets from the São Paulo retail market detected residues of oxytetracycline, florfenicol, and enrofloxacin, marking the first report of such compounds in tilapia from the Brazilian market. Although the residue levels were within Brazilian regulatory limits, enrofloxacin could to induce antimicrobial resistance due to its persistence in the environment [66]. Similarly, studies in the United States and Vietnam have shown that even sub-regulatory levels of antibiotics in seafood can contribute to developing resistant bacterial strains [67,68]. In Vietnam, for instance, shrimp samples containing antibiotic residues were found to harbor multidrug-resistant strains of non-typhoidal *Salmonella* and *Vibrio* spp., further emphasizing the need for stricter control and monitoring of antibiotic use in aquaculture [68].

Therefore, while antibiotics play a critical role in managing bacterial infections in aquaculture, their usage must be carefully regulated and monitored to prevent adverse environmental impacts and the development of antibiotic-resistant bacteria. The presence of antibiotic residues in aquatic environments and seafood products underscores the importance of adopting responsible and sustainable practices in aquaculture. International organizations like the World Health Organization (WHO), the FAO, and the Codex Alimentarius Commission, a joint initiative of the FAO and WHO, work in concert to create a cohesive global framework that safeguards both human health and the environment from the risks associated with antibiotic use in aquaculture [69,70,71]. Nevertheless, each country has its own regulations on the approval of antibiotics, usage practices, and residue limits for aquaculture products. Still, it is imperative that individual countries enforce stringent regulations tailored to their specific aquaculture practices.

### 3.2. Mechanisms of Antibiotic Resistance: The Case of Biofilms

**Figure 1:**
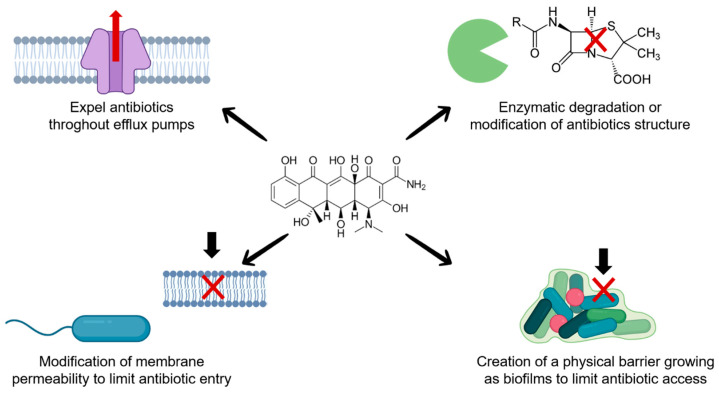
Molecular mechanisms employed by bacteria to resist antibiotic action.

**Figure 2:**
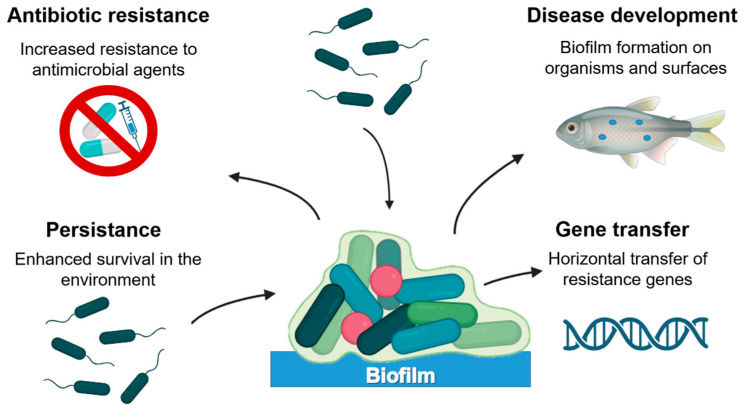
Impact of biofilms in aquaculture.

Aquatic and other bacteria can acquire antibiotic resistance through different molecular mechanisms (Figure 1) and genetic factors, which can act individually or in combination to confer survival advantages under antimicrobial pressure [72]. In aquaculture systems, these mechanisms are continuously presented due to recurrent disease outbreaks, prophylactic antibiotic use, and the open nature of aquatic environments. Specifically, biofilms represent one of the most persistent and challenging factors because they impact aquaculture at multiple levels (Figure 2) [73]. Biofilms are structured microbial communities formed in biotic and abiotic surfaces in which, bacteria are embedded in a self-produced matrix of extracellular polymeric substances (EPS), composed of polysaccharides, proteins, lipids, and extracellular DNA [9]. In production systems, this EPS matrix allows bacteria to persist on farm infrastructure (tanks, pipelines, nets, sediments) and on animal-associated surfaces [10,64]. The formation of biofilms is a regulated and dynamic process that occurs in several stages. First, planktonic cells attach reversibly to a surface, then, adhesion becomes irreversible through the production of adhesins and EPS, next, the cells proliferate and form microcolonies. Over time, the biofilm matures into a three-dimensional structure with nutrient gradients and water channels that allow internal communication and waste removal. Finally, in the dispersion stage, some cells regain motility, detach from the matrix, and colonize new surfaces to restart the cycle [74]. This dispersal stage is particularly critical from a management perspective, as it enables rapid recolonization and disease recurrence shortly after antibiotic treatment ends.

In the initial stages of adhesion, bacteria must reach the surface, aided by extracellular structures such as flagella, type I and IV fimbriae, and curli, which facilitate bacterial movement, including flagellar swimming, swarming, and twitching motility. Motility is a crucial trait in the lifestyle of *Vibrio* spp., enabling them to adapt to both aquatic environments and host-associated niches. Flagellar motility, in particular, is fundamental for various cellular processes, including movement, surface colonization, adhesion, biofilm development, and the expression of virulence factors [75]. Several studies have focused on controlling pathogenic *Vibrio* infection by inhibiting biofilm formation and attenuating the QS system. QS is an intercellular communication mechanism that enables bacteria to regulate the expression of survival and virulence genes in a cell density–dependent manner [76]. A variety of aquatic pathogens, including *Edwardsiella*, *Vibrio*, and *Aeromonas* spp., depend on QS to coordinate collective behaviors and activate virulence factors [77]. In aquaculture, rapid bacterial population growth accelerates QS activation, leading to synchronized virulence expression and biofilm maturation.

Once established, biofilms represent a major obstacle to treatment and disease control. The complex biofilm matrix allows bacteria to survive under hostile conditions, including fluctuations in pH, nutrient deprivation, temperature changes, and, notably, exposure to antibiotics and host immune responses. Within the biofilm, bacterial cells exhibit phenotypic changes, including reduced metabolic activity and altered gene expression, which render them up to 1000-fold more resistant to antibiotics than their free-living (planktonic) counterparts [11]. This is why antibiotic susceptibility observed under laboratory conditions often fails to translate into therapeutic success at the farm level.

The biofilm-associated resistance is attributed to the EPS matrix’s ability to hinder antibiotic penetration and sequester reactive molecules, effectively neutralizing antimicrobial action. In addition, this environment supports the survival of persister cells, metabolically dormant variants that tolerate higher antibiotic concentrations and can repopulate the biofilm once treatment is discontinued [78]. Persistent cells may also enhance efflux pump activity, lowering intracellular antibiotic concentrations and reducing antimicrobial efficacy [72]. Moreover, biofilms serve as reservoirs for antibiotic-degrading enzymes such as β-lactamases, aminoglycoside-modifying enzymes, and carbapenemases, which accumulate within the matrix and inactivate antibiotics before reaching their targets [79]. These localized resistance mechanisms are especially problematic in aquatic environments, where antibiotic residues accumulate in water and sediments and continuously interact with biofilm-associated bacteria.

The structural complexity of biofilms, including matrix composition and three-dimensional organization, generates gradients of oxygen and nutrients that strongly influence bacterial physiology [11]. Cells located in deeper biofilm layers exhibit slower growth rate and reduced protein synthesis, conditions that decrease susceptibility to antibiotics targeting cellular processes [74]. Such physiological heterogeneity is exacerbated by fluctuating environmental parameters (e.g., temperature, oxygen availability), further reducing treatment predictability.

Within biofilms, antibiotic resistance genes (ARGs) are efficiently transferred via horizontal gene transfer mechanisms. The close proximity of cells in the biofilm matrix favors the exchange of mobile genetic elements including plasmids, transposons, and integrons carrying resistance determinants [80]. This promotes the emergence of multidrug-resistant populations that can persist within production systems and disseminate beyond farm boundaries into surrounding aquatic ecosystems, posing risks to environmental and public health [81,82]. In *A. hydrophila* isolated from septicemic carps, a clear association between biofilm-forming capacity and antibiotic resistance profiles was demonstrated. The 19 *A. hydrophila* isolates produced biofilm, with 79% categorized as moderate producers and 21% as strong producers. Strong biofilm-forming strains also exhibited resistance to 75% of the tested antibiotics, whereas moderate biofilm producers displayed variable susceptibility patterns. A significant correlation was observed between biofilm production level and resistance to tetracycline, oxytetracycline, and amoxicillin [23]. Similar associations have been reported for *Vibrio* spp. and other aquaculture pathogens. These findings highlight that biofilm-associated resistance is not an abstract molecular phenomenon but a key driver of therapeutic failure and production losses.

Consequently, effective management strategies must extend beyond conventional antibiotic treatments to include complementary approaches that target biofilm formation, virulence regulation, and bacterial persistence. In this context, natural compounds have gained significant attention due to their capacity to interfere with QS, disrupt EPS synthesis, and enhance antibiotic penetration, offering promising synergistic strategies to reduce antibiotic dependence and delay resistance emergence [9,12].

## 4. Natural Compounds as Antibacterial and Antibiofilm Agents for Use in Aquaculture

Natural antibacterial agents include secondary metabolites from plants, marine organisms, bacteria, and fungi. These compounds are commonly classified as terpenes, steroids, alkaloids, phenolic compounds (phenolic acids, flavonoids, tannins, coumarins, and lignans), polyketides, and peptides [83]. Among them, plant-derived terpenes and phenolic compounds have been extensively reported as potent antibacterial and antibiofilm agents [9,84].

### 4.1. Plant-Derived Antibacterial Compounds

Since ancient times, plants have been widely used in traditional medicine for the treatment of various diseases, demonstrating their bioactive potential, which is linked to the complex composition of their tissues. They constitute an abundant and diverse source of secondary metabolites, including alkaloids, tannins, terpenoids, phenolic acids, and polyphenols, which can be extracted from various plant parts, such as flowers, seeds, bark, leaves, or roots [85]. These compounds not only play essential ecological roles in plant defense against pathogens and environmental stresses but have also gained considerable attention for antimicrobial applications due to their natural abundance, favorable safety profile, and remarkable structural diversity [86].

Among the wide range of plant metabolites, phenolic compounds are among the most abundant and structurally diverse groups. They are characterized by the presence of one or more aromatic rings bearing one or more hydroxyl groups, which largely determine their chemical reactivity and biological activity, and are generally divided into several major classes, including phenolic acids, flavonoids, tannins, terpenes, and terpenoids. Phenolic acids are compounds that possess an aromatic ring, with one or more hydroxyl (–OH) groups and one carboxylic acid (-COOH). Depending on the number and position of –OH groups on the aromatic ring, different phenolic acids can be distinguished, each with unique chemical and biological properties. These compounds are broadly classified into two main groups: hydroxybenzoic acids (such as gallic, protocatechuic, vanillic, and benzoic acids) and hydroxycinnamic acids (such as caffeic, ferulic, and p-coumaric acids) [87].

Flavonoids consist of a large group of polyphenolic compounds that contain a 15-carbon C6-C3-C6 backbone that forms two aromatic rings (named A and B), which are interconnected by a three-carbon bridge that forms a closed pyran ring (C ring). This core structure enables a variety of chemical modifications, resulting in numerous subclasses of flavonoids, including flavones, flavanones, flavanols, flavonols, isoflavones, and anthocyanidins [88]. Whereas, stilbenes consist of two aromatic rings connected by an ethylene bridge (C=C). Around 5000 flavonoids are known and are components of different edible parts of plants, fruits, and vegetables, cereals, tea, seasonings, among others. For example, flavonols like quercetin and kaempferol are commonly found in onions, apples, broccoli, and grapes [89,90], whereas the flavanols catechin and epigallocatechin gallate are found in green tea, apples, and cocoa [90]. According to the Phenol-Explorer database [91], the foods richest in polyphenols (among 452 sources analyzed as reported by Li, Zogona [92]) include certain spices such as cloves (15,188 mg/100 g), dried peppermint (11,960 mg/100 g), star anise (5460 mg/100 g), and Mexican oregano (2319 mg/100 g); seeds such as celery seed (2094 mg/100 g) and flaxseed meal (1528 mg/100 g); fruits like black chokeberry (1756 mg/100 g) and black elderberry (1359 mg/100 g); as well as dark chocolate (1664 mg/100 g) and cocoa powder (3448 mg/100 g).

Besides phenolic compounds, terpenes and terpenoids have demonstrated potent antibacterial activity. Terpenes constitute a large group of plant secondary metabolites built from different quantity of isoprene units (C_5_H_8_), leading to the formation of monoterpenes (limonene, myrcene α-pinene), sesquiterpenes (β-caryophyllene, farnesene), diterpenes (phytane), and triterpenes (squalene) [93]. Terpenoids are oxygenated derivatives that often exhibit stronger biological activity; some examples include carvacrol, thymol, eugenol, linalool, geraniol, eucalyptol, citral, and ursolic acid, among others. These compounds are commonly found in aromatic plants such as oregano, clove, thyme, cinnamon, lavender, lemongrass, eucalyptus, etc. [94]. Monoterpenoids are major constituents of many essential oils (EOs); which are non-water-soluble mixtures rich in volatile aromatic compounds. Typically EOs owe their characteristic fragrance and biological properties to their monoterpenoid content and are important to plants for communication, to attract pollinating insects and to repel predators [93]. The structural diversity of plant-derived compounds not only determines their physicochemical properties but also underlies a wide range of biological activities. Among these, the antibacterial activity has been one of the most extensively studied and recognized, and has been tested against several human and animal pathogens, including those affecting aquaculture organisms (Table 3). Natural compounds from plants possess different bioactive properties, such as anticancer, antiinflammatory, antimutagenic, antiviral, antifungal, antibacterial, antibiofilm and antivirulence.

Several compounds have been shown to significantly inhibit biofilm formation and interfere with intercellular communication systems. For example, resveratrol at 100 µg/mL reduced biofilm formation of *A. hydrophila* by 50.66% and significantly reduced the swimming and swarming motility [113]. On the other hand, the study reported by Vazquez-Armenta, Aros-Corrales [84] evidenced the antibiofilm and antivirulence potential of the phenolic compounds quercetin, morin, vanillic acid, and protocatechuic acid, against two strains of *V. parahaemolyticus*, a human pathogenic (Vp320) and one isolated from shrimp (Vp124). Results showed that subinhibitory concentrations (0.125–0.5 × MIC) reduced biofilm biomass by 63.22–92.68%. In addition, the flavonoids quercetin and morin inhibited the motility of both *Vibrio* tested strains (15.86–23.64% for Vp124 and 24.28–40.71% for Vp320), being morin the most effective compound. These indicate that an interruption in the QS system has an impact on biofilm inhibition and reduced virulence.

The development of more sustainable aquaculture is an urgent need, as it provides an important source of aquatic products for human consumption. One of the concerns associated with the use of alternative compounds to the already widely used and tested antibiotics is the possible negative effects that these may have on farmed species. Nevertheless, several studies have evidenced that the use of plants, for example, as feed additives, provides beneficial effects to farmed organisms (Table 4). Even the FDA has recognized phytochemicals as Generally Recognized as Safe (GRAS). Among the most well- documented biological effects of phytochemicals on marine organisms are immunostimulant, appetite-stimulating, antioxidative, anti-inflammatory, and antimicrobial activities [114]. For example, a study evaluated the effect of *Origanum vulgare* EO in the culture of *Cyprinus carpio* L. The EO was administered in a diet of 0–20 g/kg, and it was observed that after eight weeks, it significantly increased the activity of the antioxidant hepatic enzymes superoxide dismutase and catalase. Additionally, the malondialdehyde was decreased, and the expression of interleukin-1β and interleukin-10 genes were upregulated. These effects were associated with significantly lower mortality associated with pathogenic bacteria *A. hydrophyla* [30]. Similarly, *Citrus limon* EO was used to improve fingerlings’ (*Labeo victorianus*) biochemical and haemato-immunological parameters. Remarkably, blood and immune cells as serum immunoglobulins increased as dietary inclusion of *C. limon* EO increased from 1 to 5%, resulting in lower mortality of fingerlings challenged with *A. hydrophyla* [115].

### 4.2. Bioactive Molecules from Marine Organisms

The marine ecosystem is a rich yet largely unexplored reservoir of bioactive compounds with antimicrobial and antivirulence potential. These secondary metabolites arise from the intense biological competition for space, predation, and the dynamic tidal forces characteristic of this environment [116]. A wide variety of marine organisms, such as bacteria, fungi, invertebrates, and algae, can be explored as valuable sources of antimicrobial compounds with promising applications in aquaculture [117]. For instance, sessile marine organisms such as sponges, algae, and corals have evolved survival mechanisms to defend themselves against predators by activating complex secondary metabolic pathways that produce potent bioactive substances [117]. It has been reported that marine sponges harbor high microbial abundance, with diverse communities including bacteria, fungi, archaea, and viruses. Several studies have demonstrated that bioactive compounds found in sponges are actually produced by the microbial communities in symbiosis with them [118,119].

**Table 4:** Health beneficial effects of natural compounds in aquaculture organisms.

Natural Compound/Source	Dose	Cultured Organism	Treatment Administration	Observed Health Effects	Reference
Date seed EO	0.5–2.0 mL/kg	Nile tilapia (*Oncorhynchus mykiss*)	The EO was incorporated into the diet and fished were fed in morning and midafternoon for 45 days.	The treatment reduced the feed conversion ratio and the fishes showed an increase in weight gain.	[120]
*Morus alba*, *Curcuma xanthorrhiza*, and *Boesenbergia rotunda* extract	50, 100, 150, and 200 mL/kg	Shrimp (*Penaeus monodon*)	The extract was incorporated by spying on surface pellets and shrimp were fed at 07:00, 12:00, 17:00, and 22:00 h.	Fermented extracts caused a 100% survival rate and the treatment of 100 mL/kg significantly increased the shrimp weight gain.	[121]
Oregano EO	0.3 g/kg	Grouper (*Epinephelus fuscoguttatus*)	EO was incorporated into pellet formulation and then fish were slowly hand-fed at 7:00 a.m. and 7:00 p.m. during 8 weeks.	Increased the abundance of Firmicutes and Baiteroidetes in intestine, activate antioxidant defense system, and enhance the immunity via acid phosphatase, lysozyme, and complement C3.	[122]
	0.1, 0.2, and 0.4%	Catfish (*Pangasionodon hypophthalmus*)	Oregano EO was incorporated on the surface of the pellets and the organisms were fed by hand four times daily, at 07:00, 11:00, 15:00, and 20:00 h.	The oregano-based diet improved the nutrient absorption and 0.1% oregano EO supplemented fed protected the fish from *A. hydrophila* infection.	[123]
*Lagenaria siceraria* extract	2.5, 5, and 10 mL/L	Nile tilapia (*Oreochromis niloticus*)	Organisms were fed twice daily (at 9:00 and 14:00) for 60 days.	Reduced the mortality rate of challenged organisms with *A. hydrophila,* increased red and white blood cells and hemoglobin, while hepatic enzymes and glucose levels were significantly lower.	[124]
Carvacrol	3 g/kg	Nile tilapia (*Oreochromis niloticus*)	Carvacrol was included in the diet by spraying. Treatment was applied twice daily for 1 month, then challenged with *Cryptococcus uniguttulatus* (10^8^ CFU/mL) and continued with the supplemented feeding, and the second group was supplemented on the second day post challenge.	Treated fish showed a relative percent survival of 90%, and enhanced growth performance. Myeloperoxidase activity and total immunoglobulins significantly increased compared to non-supplemented fish.	[125]
	1.0 g/kg	Grass carp (*Ctenopharyngodon idella*)	The feeding was performed twice a day, daily at 9 a.m. and 5 p.m. for 1 week, then were challenged with 200 µL of *A. hydrophila* (5.0 × 10^7^ CFU/mL).	The treatment protected the carps from *A. hydrophila* infection. The survival rate was 56% compared with 24% positive group.	[126]
	20 μg/mL	Shrimp (*Penaeus vannamei*)	The organisms were feed with commercial diet coated with the oil at four times per day.	Carvacrol enhanced the growth performance of shrimp, reduced the feed intakes, increase the number of total haemocyte counts and lysozyme activity.	[111]

Actinobacteria have been recognized as producers of several antibacterial molecules; for example, some *Streptomyces* strains have shown inhibitory effects against *A. sobria*, *A. hydrophila*, and *E. tarda*. Some virulent *Vibrio* strains (*V. alginolyticus*, *V. parahaemolyticus*, and *V. harveyi*) were sensitive to soil isolates of Actinobacteria. Similarly, Raissa, Waturangi [127] reported that Actinomycetes isolates extracts at concentration of 20 mg/mL inhibited the growth of *V. harveyi* and *S. agalactiae*. In addition, the isolates reduced biofilm formation of *A. hydrophila*, *V. harveyi*, and *S. agalactiae* by 85.11, 44.23, and 53.42%, respectively. A recent study reported that saponins isolated from the sea cucumber *Holothuria leucospilota* exhibited antimicrobial activity against *A. hydrophila*, with a MIC and MBC of 30 and 80 µg/mL, respectively. At sub-MIC levels, these saponins significantly downregulated the QS regulators ahyI and ahyR, resulting in a ~87% reduction in biofilm formation compared to untreated controls and notable decreases in key virulence factors such as hemolytic, protease, and lipase activities. Additionally, treated cells showed impaired swarming motility, indicating disruption of behaviors linked to pathogenicity [128]. On the other hand, the peptide YM-266183 isolated from the marine sponge *Halichondria japonica* significantly inhibited the growth of *S. aureus* and *Enterococci* at 0.68 and 0.025 µg/mL, respectively. Another study reported the isolation of the compound viridicatol from the Arctic endophytic fungus *Penicillium* sp. Z2230. These compounds showed antivibrio activity, inhibiting the growth of *V. parahaemolyticus*, *V. cholerae*, *V. vulnificus*, and *V. alginolyticus* at MIC values of 63.2 µg/mL for the first three bacteria and 126.4 µg/mL for *V. alginolyticus*. In addition, docking analysis revealed that this compound interacts with Vibrio’s peptide deformylases, key enzymes in protein synthesis and bacterial survival [129]. On the other hand, algae also have been proved as a source of antibacterial molecules, for example, low molecular weight phlorotannins extracted from brown algae *Sargassum thunbergii* (900 µg/mL) inhibited the growth of *V. parahaemolyticus* and damaged cell membrane and cell wall damage [130].

## 5. Synergistic Interactions Between Natural Compounds and Antibiotics

There are several ways in which antimicrobial resistance can be prevented, reduced, or reversed, and using plant extracts with intrinsic antimicrobial properties has proven to be a promising method. Evidence suggests that plant-derived compounds can enhance the efficacy of conventional antibiotics through a synergistic interaction, demonstrating the potential of such combined therapies in combating resistant bacterial strains [131,132]. Natural compounds of diverse origins have been shown to possess antimicrobial activity against pathogenic bacteria as previously showed. Among these, phytochemicals became the most studied compounds in combination with antibiotics. Evaluating antimicrobial interactions has become increasingly important due to the rising prevalence of antibiotic-resistant bacteria [95]. The need for synergy testing methods to identify effective combinations for antimicrobial therapy is driven by the goals of broadening the antimicrobial spectrum, reducing antibiotic concentrations, minimizing toxicity and environmental impact, and, not less importantly, decreasing the likelihood of developing bacterial resistance [133]. There are several methods to study the synergistic interactions between antibiotics and natural compounds, being the most common the in vitro time-kill assay, gradient diffusion method (direct overlay, cross, MIC:MIC ratio), the E-test and checkerboard assay [134].

The time-kill assay determines the reduction in the viable bacterial counts after exposure to combined and single antimicrobials. The assay is performed in culture broth containing the antimicrobials and the bacterial inoculum, and colony counts are taken at different time intervals (commonly every two hours) during 24 h and subcultured on agar plates. Synergism is considered as ≥2 log CFU/mL reduction in the bacterial growth exposed to the combination compared to the most active single antimicrobial. In contrast, antagonism is when ≥2 log CFU/mL of bacterial growth is present in the combined antimicrobial treatment. The effect is interpreted as indifferent when the difference is <2 log CFU/mL [135]. On the other hand, in the E-test, two reactive strips containing a concentration gradient of the two antimicrobials tested are placed perpendicularly (intercepting at the MIC values) on an agar plate previously inoculated with the bacteria. The inhibition zones should be in the lower right quadrant if the compounds showed synergy. In contrast, for an antagonistic effect, the bacteria will be inhibited in the upper left quadrant (where the higher concentrations of the antimicrobials are placed) [136].

**Figure 3:**
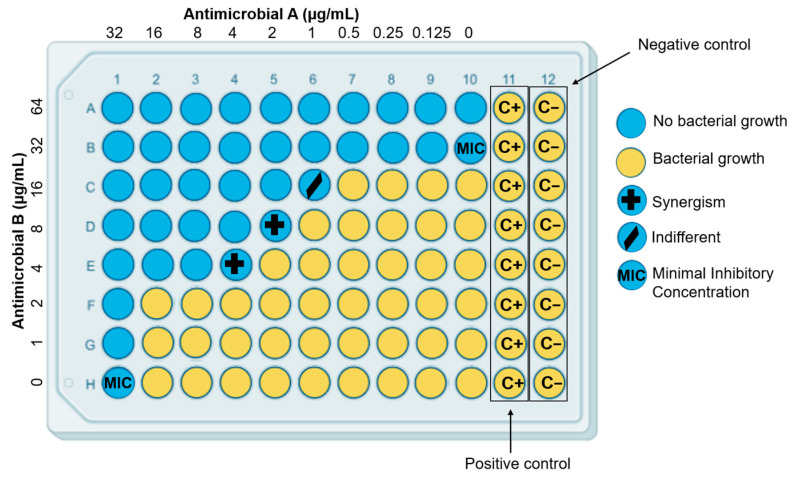
Checkerboard assay for antimicrobial synergy evaluation.

The checkerboard assay is one of the most popular and used methods; it combines different concentrations of two antimicrobials in a microplate and observes the inhibitory effect on bacterial growth. This method involves testing different concentrations of the two agents in combination, based on their MIC values, to determine their combined effect on microbial growth (Figure 3). The synergy is evaluated throughout determining the fractional inhibitory concentration index (FICI). The FICI is the standard reference parameter to quantify drug interactions in antimicrobial research. To calculate this index, the Fractional Inhibitory Concentration (FIC) of the tested drugs is determined by the following formulas:

(1) $${\mathrm{FIC}}_{A}=\frac{\mathrm{MIC}\;\mathrm{of}\;\mathrm{drug}\;A\;\mathrm{in}\;\mathrm{combination}}{\mathrm{MIC}\;\mathrm{of}\;\mathrm{drug}\;A\;\mathrm{alone}}$$

(2) $${FIC}_{B}=\frac{MIC\;of\;drug\;B\;in\;combination}{\mathrm{MIC}\;\mathrm{of}\;\mathrm{drug}\;B\;\mathrm{alone}}$$

The sum of FIC_A_ and FIC_B_ provides the FICI value (FICI = FIC_A_ + FIC_B_), which indicates the degree of interaction between the two drugs. Synergy is considered when FICI is ≤0.5, indifferent between >0.5 and ≤4, and antagonism > 4. “Synergy” means that the antimicrobial effect of the combination is higher than the sum of the antimicrobial effects of the compounds individually. An interaction between antimicrobials is “indifferent” when the combination of two compounds has no increase in inhibitory activity or a slight increase, and is antagonistic if the effect is smaller than that of the individual antimicrobials [137].

The synergistic combination of antibiotics and phytochemicals represents a promising strategy to reduce antibiotic resistance in aquaculture settings. Synergistic interactions, commonly defined by FICI values ≤ 0.5, are particularly relevant because they enable effective bacterial control at sub-inhibitory antibiotic concentrations. This reduction in antibiotic dosage may decrease selective pressure for resistance development and limit the accumulation of antibiotic residues in aquatic environments.

Some natural compounds have proved to inhibit the viability of antibiotic-resistant bacteria directly, whereas others can sensitize bacteria or inhibit virulence factors, reversing the resistance [138] (Table 5). In the study reported by Lu, Tsui [139], the impact of combination treatments was clearly demonstrated through a substantial reduction in the MIC value of the β-lactam antibiotic carbenicillin when used in combination with *Gracillaria* sp. extracts. Specifically, against *V. parahaemolyticus*, the MIC of carbenicillin (256 µg/mL) was reduced by 4- to 16-fold compared to the antibiotic alone. A similar trend was observed for *V. cholerae*, for which the MIC (8 µg/mL) was reduced by at least twofold. Similarly, in the study reported by Zhao, Cui [140], the combination of florfenicol with the flavonoid quercetin resulted in a 32-fold reduction in the MIC (from 360 to 0.0078 µg/mL) of the antibiotic against *A. hydrophila*, yielding an FIC index of 0.28, which indicates a synergistic interaction between both compounds. Additionally, this combination reduced in vitro bacterial viability by 16.3- to 191.4-fold compared with florfenicol alone. Importantly, these findings were further validated in vivo to assess their potential practical application in aquaculture. The combined treatment significantly reduced the bacterial load of *A. hydrophila* in the liver, spleen, and kidney of common carp, achieving reductions of 610.6-fold compared with the antibiotic treatment, and resulted in a survival rate of 90%, compared with 30% and 20% for florfenicol and quercetin alone, respectively, and 10% in the untreated control group.

On the other hand, a study reported by Mascarenha, Jo [149] evaluated the synergistic antibacterial activity of a ethyl acetate and methanolic extracts of the brown seaweed *Eisenia bicyclis* in combination with erythromycin and oxytetracycline against *E. tarda*, *V. harveyi*, and *Photobacterium damselae*. Results evidenced that the combination of the ethyl acetate extract-erthromycin significantly enhanced the antibacterial activity by reducing 2–4 times the MIC values of both compounds against the tested pathogens [149]. However, although this study provides valuable evidence of synergistic activity, it is important to consider the limitations associated with the use of crude extracts and fractions. Such matrices contain complex mixtures of bioactive compounds whose qualitative and quantitative composition may vary substantially depending on environmental factors, growth conditions, harvest time, and extraction procedures [150]. This inherent variability can compromise reproducibility and hinder the standardization required for practical aquaculture applications.

The study by Guo et al. (2021) [145] (Table 5), evaluated the combined therapy of oxytetracycline with tea polyphenols. A highly synergistic activity was observed when combined 1/64 MIC of tea polyphenols (0.002437 mg/mL) and 1/8 of oxytetracycline (1 µg/mL), resulting in a FICI of 0.14. Additionally, it was shown that this treatment enhances the activity of immune-related enzymes (alkaline phosphatase and lysozyme) of *E. carinicauda* infected with *V. parahaemolyticus*, as well as the expression of immune-associated genes. Nevertheless, the evaluation was limited to a 24 h period, which restricts a comprehensive assessment of the durability of the enhanced resistance, the temporal stability of immune parameters, and the sustained efficacy of the treatment under longer-term culture conditions. It is important to mention that some studies have evaluated the application of combined therapies in vivo, demonstrating their biological impact and supporting the effects previously observed in vitro. However, their assessment has generally been limited to short experimental periods.

Similarly, it was evaluated the in vivo effect of quercetin-florfenicol combination as a protective therapy on AHPND infected *L. vannamei* shrimps. Results showed that the low (200 mg/kg quercetin–7.0 mg/kg florfenicol), moderate (400 mg/kg quercetin–15 mg/kg florfenicol) and high (800 mg/kg quercetin–30 mg/kg florfenicol) combined dosses reduced (*p* < 0.05) the cumulative shrimp mortality and showed lower *Vibrio* density with 65.80–76.94% at 1 day postinfection, compared to florfenicol and quercetin alone (52.27–58.59%). At the 5 day postinfection, the moderate and high concentration combined groups showed a complete clearance of *Vibrio*. Additionally, the activity of immune-related enzymes glutathione peroxidase, superoxide dismutase, and lysozyme significantly increased compared to infection, quercetin and florfenicol only groups. It is important to mention that a lower affectation of hepatopancreatic tubules were observed in the combined treatment in the histological assay. This means that the mere inclusion of quercetin in antibiotic treatment improves hepatopancreatic integrity better than traditional florfenicol treatment [151]. Although the study clearly established the synergistic benefits of the florfenicol-quercetin combination, a valuable avenue for future research will be evaluate the long-term effects of this combined treatment beyond the 5 days of the experiment and the post-recovery state of shrimp. These findings provide a strong theoretical and empirical basis for further translate these in vivo laboratory evaluation to commercial aquaculture settings. The pronounced reductions in the MIC values of the antibiotic component not only reflect improved antimicrobial potency but also suggest a practical strategy to minimize antibiotic input, potentially reducing selective pressure for resistance development and mitigating adverse effects associated with high antibiotic dosages.

**Figure 4:**
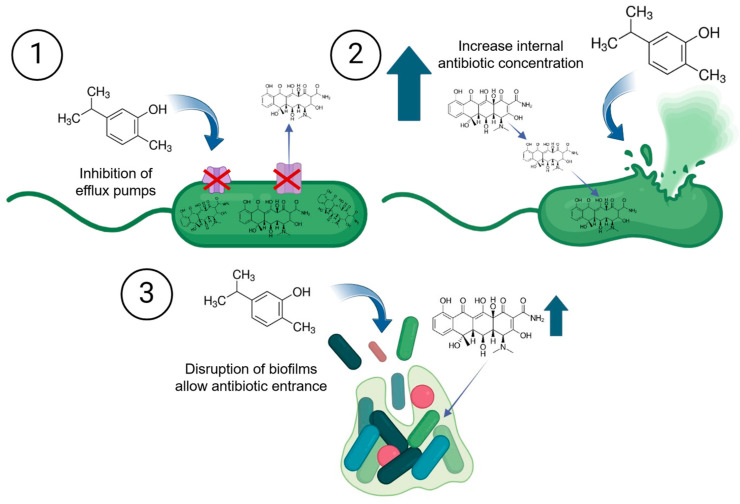
Natural compounds improve the antibiotic’s effects by increasing their concentration into bacterial cells through: (1) the inhibition of efflux pumps, (2) damage to bacterial membrane, or (3) disrupting biofilm structure.

Natural compounds can enhance the effect of aquaculture antibiotics through different mechanisms (Figure 4). They may disrupt bacterial cell walls and membranes, increasing the permeability of antibiotics and allowing them to penetrate more effectively [152]. Some natural compounds can inhibit efflux pumps, which bacteria use to expel antibiotics, thereby increasing the intracellular concentration of the antibiotic [153] or affect the activity of antibiotic-degrading enzymes. Others might target bacterial biofilms, structures that protect bacteria from antibiotic action, thereby exposing the bacteria to the antibiotics’ effects [154]. For example, the bioactive compound luteolin from *Commelina communis* inhibited the AcrB-TolC, a multidrug-resistant efflux pump of *A. hydrophila*. Flavonoids such as quercetin and catechin interacts with membrane components increasing their fluidity and permeability. This disruption of bacterial membrane facilitates the penetration off antibiotics [155]. Another example is those of epigallocatechin gallate, which caused a membrane depolarization by bind lipid bilayers and improved the oxacillin uptake in the human pathogen methicillin-resistant *S. aureus* [156].

The study reported by Lu, Tsui [139] provided an approach to the possible mechanism of action exerted in the combination of carbenicillin with the extract of *Gracillaria* sp. The results showed that the addition of the extract reduced the degradation capacity of carbenicillin and nitrocefin by the recombinant carbapenemase enzyme VarG of *V. cholerae* by 20 and 60%, respectively, allowing to maintain effective doses to exert the antibacterial effect.

On the other hand, one important factor to be considered in the evaluation of the combined therapies is the type of strains used, most studies including those reported in Table 5 are based on reference strains instead of pathogens directly isolated from aquaculture systems. This approach helps to ensure reproducibility, but limits the extrapolation of the results to real production conditions. Pathogens circulating in aquaculture environments are constantly interacting with the host and several environmental stressors. As consequence the bacterial population in the farms showed a high variability in terms of virulence patterns and antibiotic susceptibility. The relevance of this limitation is exemplified in the study of Vazquez-Armenta, Aros-Corrales [84], who evaluated the response of two strains of *V. parahaemolyticus* (a reference strain and one isolated from shrimp) to plant phenolics. Their results evidenced notable differences in biofilm formation and motility. It was observed that the reference strain was more susceptible to quercetin and morin, with a biofilm reduction of 85.31–91.66%, compared with 77.97–80.85% of that isolated from shrimp. With respect to motility, the flavonoids caused a motility inhibition of 23.63 and 40.71%, and 15.86 and 24.28% for the reference strain and the shrimp isolate, respectively, showing major resistance. This variability suggests that evaluating antimicrobial susceptibility only using a reference strain may not be representative of the field isolates that predominate in aquaculture systems.

Although many antibiotic–natural compound combinations do not exhibit strong bactericidal activity when applied individually, growing evidence indicates that their synergistic potential could lie in targeting bacterial virulence rather than viability. As observed previously, the concentrations used to evaluate the antibiofilm and antivirulence activity of plant-derived compounds are at sub-inhibitory concentrations. These reduced concentrations significantly disrupt biofilm architecture, inhibit EPS production, and/or interfere with the QS systems, all of which are critical determinants of bacterial persistence and antimicrobial tolerance [142,144]. Biofilms, in particular, as previously stated, act as protective niches that reduce antibiotic penetration and promote phenotypic resistance. Therefore, agents capable of dispersing mature biofilms or preventing their formation as demonstrated by plant-derived compounds, can indirectly restore antibiotic susceptibility by exposing bacterial cells to effective drug concentrations without imposing strong selective pressure for resistance development [157]. This is why combining natural-derived compounds with antibiotics and targeting biofilms is a viable and promising strategy to reduce antibiotic resistance in aquaculture. Despite the increasing interest in the application of natural compounds as a strategy to reduce antibiotic doses and counteract the development of bacterial diseases in aquaculture, scientific evidence remains limited. In recent years, numerous in vitro studies have demonstrated the antimicrobial activity of phenolic compounds, as well as their ability to interfere with the synthesis of virulence factors, biofilm formation, and other bacterial resistance mechanisms. However, most investigations have focused on the individual effects of these compounds under controlled laboratory conditions, which differ substantially from the complex, dynamic, and variable environments characteristic of aquaculture production systems.

One important point to be considered is that in many of these studies, the synergism is studied in many cases from phenotypic outcomes, rather than supported by other molecular techniques, whose limited approach limits the understanding of how antibiotic-natural compound combinations modulate the physiology and virulence of pathogenic bacteria. In this sense, target-specific assays and molecular interaction techniques could be proposed for a deeper understanding of synergism mechanisms.

## 6. Future Directions

Natural compounds have attracted significant attention due to their well-documented antibacterial and antibiofilm properties [9,12]. In recent years, the combination of these compounds with conventional antibiotics has emerged as a promising strategy to achieve synergistic effects, allowing for a reduction in effective antibiotic doses for disease control and, consequently, contributing to the mitigation of antibiotic resistance [140]. Despite the advances reported to date in natural compound–antibiotic combination therapies, several challenges remain. One of the major obstacles is the limited in vivo validation of these strategies. Most studies are restricted to in vitro assays, while critical aspects such as their impact on clinical and physiological parameters of exposed organisms, stability under aquaculture conditions, appropriate delivery systems, and potential effects on non-target microbiota remain insufficiently explored. In this context, future research should prioritize well-designed in vivo studies that integrate pharmacokinetics, toxicity, and efficacy assessments under realistic farming conditions (influence of temperature, salinity, organic matter, pond microbiota). Additionally, the development of stable formulations and efficient delivery vehicles such as encapsulation (nanoemulsions, nanoliposomes, chitosan nanoparticles), which has shown potential in improving stability and bioavailability of natural compounds in aquaculture [158]. Also, further investigation is needed into the mechanisms involved in biofilm inhibition and its association with the reduction in antibiotic resistance. Finally, evaluating the long-term ecological and microbiological impacts of these combinations will be crucial to support their safe and sustainable application in aquaculture.

## 7. Conclusions

The intensification of aquaculture has led to a higher incidence of bacterial diseases and an increased dependence on antibiotics, which has accelerated the emergence of antimicrobial resistance and the persistence of infections, particularly those associated with biofilm formation. This review highlights that natural compounds represent promising adjuvants to conventional antibiotics, as their synergistic use can enhance antibacterial efficacy, reduce the required antibiotic doses, and disrupt biofilm development, thereby improving treatment outcomes. The integration of natural compounds with antibiotics offers a sustainable and viable strategy to strengthen disease control while limiting the spread of antibiotic resistance in aquaculture systems. Nevertheless, future research should focus on in vivo validation under real aquaculture conditions to facilitate their practical application, detailed mechanistic studies, effect on organisms’ physiology, and evaluation of safety and stability. Additionally, cost-effectiveness and regulatory feasibility should be addressed. Overall, antibiotic–natural compound combinations represent a viable strategy to improve disease control while mitigating antibiotic resistance in aquaculture.

## Figures and Tables

**Table 1:** Major bacterial pathogens affecting aquaculture species.

Disease	Bacteria	Species Affected	Specific Disease/Main Symptoms
Vibriosis	*Vibrio anguillarum*	Eel (*Anguilla anguilla*), seabass (*Dicentrarchus labrax*)	Vibriosis: darkened skin, lethargy, ulcers, hemorrhages
	*Vibrio harveyi*	Shrimp larvae (*Penaeus vannamei*), grouper (*Epinephelus* sp.)	Luminescent vibriosis: erratic swimming, reduced feeding, external pale or reddish color
	*Vibrio parahaemolyticus*	Shrimp species (*P. vannamei*, *P. monodon*, *P. chinensis*)	AHPND: pale shrunken hepatopancreas, empty gut, reduced feeding
	*Vibrio vulnificus*	Eel (*A. anguilla*), tilapia (*Oreochromis niloticus*), seabass (*D. labrax*)	Hemorrhagic septicemia: hemorrhages on the skin, fins, and around the mouth or gills, ulcer lesions on the surface, lethargy
Aeromoniasis	*Aeromonas hydrophila* *Aeromonas caviae* *Aeromonas sobria*	Channel catfish (*Ictalurus punctatus*), tilapia (*O. niloticus*), rohu (*Labeo rohita*), and other cyprinids	Motile Aeromonas Septicemia: hemorrhages, abscesses, ulcerations, ascitic fluid, and anaemia
	*Aeromonas salmonicida*	Salmonids	Furunculosis: lethargy, loss of appetite, hemorrhages in musculature and internal organs, boil-like lesions in the skin, and ascites
Edwardsiellosis	*Edwardsiella tarda* *Edwardsiella anguillarum* *Edwardsiella piscicida*	Tilapia (*O. niloticus*), Freshwater catfish (*P. hypophthalmus*), eel (*A. anguilla*)	Edwardsiellosis: internal abscesses, exophthalmia, ulcerative lesions, ascites, lethargy
	*Edwarddsiella ictaluri*	Freshwater catfish (*P. hypophthalmus*), Channel catfish (*I. punctatus*)	Enteric Septicemia of Catfish
Pseudomonasis	*Pseudomonas anguilliseptica*	Eel (*A. anguilla*), tilapia (*O. niloticus*), cod (*Gadus morhua*)	Sekiten-byo in eel: hemorrhages, pale gills, exophthalmia, lethargy
	*Pseudomonas fluorescens*	Tilapia (*O. niloticus*), bighead carp (*Hypophthalmichthys nobilis*), rainbow trout (*Oncorhynchus mykiss*)	Erythroderma in carps: inflammation, bleeding from the skin, and a loss of scales
Flavobacteriosis	*Flavobacterium branchiophilum*	Rainbow trout (*O. mykiss*), Atlantic salmon (*Salmo salar*)	Bacterial gill disease: swollen, pale gills with excess mucus, respiratory distress
	*Flavobacterium columnare*	Tilapia (*O. niloticus*), cyprinids	Columnaris disease: gill necrosis, skin and fin lesions, cotton-like plaques
	*Flavobacterium psychrophilum*	Rainbow trout (*O. mykiss*), salmonids (*Salmo*, *Oncorhynchus*, *Salvelinus* spp.)	Bacterial Coldwater Disease: fin erosion, skin lesions, spinal deformities, high mortality in fry (Rainbow Trout Fry Syndrome)
Mycobacteriosis	*Mycobacterium* *fortuitum* *Mycobacterium marinum*	Tilapia (*O. niloticus*), catfish (*I. punctatus*), cyprinids, snakehead (*Channa argus*), striped bass (*Morone saxatilis*)	Fish tuberculosis: scale loss, abnormal swimming, lethargy, reduction in feeding
	*Nocardia asteroides*	Amberjacks (*Seriola* spp.)	Nocardiosis: nodules in the gills, spleen, kidney, and liver, with or without ulcers and skin nodules
Streptococcosis	*Streptococcus agalactiae*	Tilapia (*O. niloticus*), channel catfish (*I. punctatus*), rainbow trout (*O. mykiss*)	Streptococcosis: exophthalmia, hemorrhages in the brain and eyes, erratic swimming, high mortality
	*Streptococcus iniae*		Streptococcosis: meningoencephalitis, eye opacity, lethargy, spinal deformities
Renibacteriosis	*Renibacterium* *salmoninarum*	Salmonids (*Salmo*, *Oncorhynchus*, *Salvelinus* spp.)	Bacterial Kidney Disease (BKD): swollen kidneys, anemia, internal granulomas, lethargy
Other diseases	*Hepatobacter penaei*	Penaeids (*Penaeus* spp.)	Necrotising hepatopancreatitis: darkened or black gills, atrophied and discolored hepatopancreas, empty gut, lethargy, soft shell

**Table 3:** Antibacterial and antibiofilm activity of natural compounds against aquaculture pathogens.

Compound/Source	Dose	Bacteria	Effect	Reference
Plant extract/Essential oil				
*Eugenia caryophyllus* EO + *Trans*-cinnamaldehyde	0.01 mg/mL	*Aeromonas species*	Showed synergistic inhibitory activity at concentrations of 0.125 × MIC of *E. caryophyllus* and 0.25 × MIC of *trans*-cinnamaldehyde.	[95]
*Mentha piperita* EO	0.0035 mL	*V. parahaemolyticus*	Showed a growth inhibition diameter of 18.20 mm.	[96]
Thyme, oregano and tea tree EO	10 µL	*A. salmonicida*	The EOs showed inhibition halos of 32.00–43.67, 33.33–46.67, and 21.33–39.00 mm for thyme, oregano, and tea tree, respectively, against 12 isolates from rainbow trout. Oregano and thyme EOs were the most effective, inhibiting biofilm formation at 0.0078 µL/mL while thyme EO did so at 0.015 µL/mL.	[97]
		*A. veronii*	Thyme and tea tree EOs showed maximum inhibition halos of 43.00 and 39.33 mm, respectively, whereas for biofilm inhibition they were 0.03 and 0.06 µL/mL.	
*Aloe vera*	50–250 mg/L	*E. tarda*	*Aloe vera* inhibited the growth (50 mg/L) and showed inhibition halos (4.91–6.01 mm) with increasing concentrations of the extract.	[98]
*Chaetomorpha antennina*	50, 100, 150 and 200 µL	*V. parahaemolyticus*	The extract concentrations caused inhibition diameter halos of 17, 21, 28, and 36 mm, respectively.	[99]
Isolated compounds				
*Trans*-cinnamaldehyde	0.01 mg/mL	*A. salmonicida* *A. sobria*	Inhibited the growth of both strains and their effect was comparable to those of oxytetracycline and higher compared to gentamicin.	[95]
Eugenol	0.1–0.6%	*V. parahaemolyticus*	Eugenol at 0.1% reduced 3 and 2.5 log of CFU/cm^2^ the biofilm cells of clinical and environmental isolates and at 0.4% more than 4.5 and 4 log CFU/cm^2^ on crab surfaces. At 0.6% the biofilms were reduced below the detection limit.	[100]
	0.2 mg/mL	*V. vulnificus*	Eugenol inhibits the growth of *V. vulnificus* by disrupting cell membrane integrity through oxidative stress, leading to leakage of intracellular components, while also demonstrating significant efficacy in the removal of this pathogen	[101]
Citral	40 μL/L	*V. vulnificus*	Citral induces *V. vulnificus* to enter the VBNC state.	[102]
	0.125 mg/mL	*V. parahaemolyticus*	Citral (at 0.25 and 0.5 × MIC) reduced 17.57% and 32.33% of biofilm formation. Additionally, it inhibited other virulence factors such as motility, and extracellular polysaccharide production.	[103]
	0.125 mg/mL	*V. alginolyticus*	Citral inhibited the production of virulence factors and reduced fish infection by repressing the genes involved in the quorum sensing system.	[104]
Quercetin	27.5–110 μg/mL	*V. parahaemolyticus*	0.125–0.5 × MIC values reduced the biofilm formation by 0.68, 1.43, and 3.70 log CFU/cm^2^, as well as by 0.74, 1.40, and 3.09 log CFU/cm^2^ on shrimp and crab surfaces, respectively. Additionally, the biofilm formation (*vp0952* and *vp0962*) and flagella motility (*flaA* and *flgL*) related genes were down-regulated at the same concentrations.	[105]
6-aminoflavone 3,2-dihydroxyflavone and 2,2-dihydroxy-4-methoxybenzophenone	20 and 50 µg/mL	*V. parahaemolyticus*	Planktonic growth was inhibited at 20 and 50 µg/mL and the compounds inhibited preformed biofilms by 2.1–2.8 log CFU/cm^2^ at 100 µg/mL and affected bacteria aggregation and motility.	[106]
Carvacrol	0.0781–0.1563 mg/mL	*V. parahaemolyticus* *V. alginolyticus* *V. harveyi*	The MIC values of carvacrol were 0.0781 mg/mL for *V. alginolyticus* and *V. harveyi* while for *V. parahaemolyticus* it was 0.1563 mg/mL. In addition, carvacrol (15 µL) generated inhibition halos of 9.63, 12.92, and 13.75 mm, respectively.	[107]
Gallic, vanillic, and protocatechuic acids Rutin Quercetin Morin	0.8 to 35.03 mM	*V. parahaemolyticus*	Inhibited the bacterial growth and at 0.125–0.5 × MIC reduced biofilm formation by 63.22–92.68%.	[12]
Quercetin	500 µg/mL 8 to 64 mg/mL	*A. hydrophila*	Quercetin inhibited bacterial growth. Biofilm production was reduced by 46.3% compared with streptomycin (71.8%).	[108]
	2500 µg/mL 312 µg/mL	*S. iniae* *E. tarda*	Growth was inhibited at MIC values.	[109]
Vanillic acid	1 mg/mL	*V. alginolyticus*	Inhibited the growth and at sub-MIC reduced biofilm formation, exotoxin production, motility, and reduced the expression of the virulence genes *fliK*, *lafA*, *ypG*, *lafK*, *fliS*, *asp*, and *luxR*.	[110]
Thymol	20 μg/mL	*V. harveyi* *V. parahaemolyticus*	Thymol enhanced the growth performance of shrimp, reduced the feed intakes, increased the number of total haemocyte counts and lysozyme activity.	[111]
	150 μg/mL	*V. parahemolyticus*	Thymol induces Fenton-reaction-dependent ferroptosis. Proteins involved in ROS production, lipid peroxidation, and DNA repair were significantly upregulated following thymol treatment. Thymol promotes the release of Fe^2+^ from ferritin proteins at amino acid residues H46 and F42.	[112]

CFU: colony forming units; MIC: minimal inhibitory concentration; VBNC: viable but non culturable.

**Table 5:** Antibacterial and antibiofilm activity of the combination of aquaculture antibiotics with naturally derived compounds.

Antibiotic	Natural Compound/Extract	Bacteria	Effect	Reference
Fenicols				
Florfenicol	*Lippia sidoides* and *Cymbopogon citratus* EO	*Aeromonas hydrophila*	The MIC values of *L. sidoides* and florfenicol were reduced by 4–16- and 3.8–7.8-fold, respectively, resulting in synergistic FICI values of 0.25 and 0.18.	[141]
Florfenicol	Rutin	*Aeromonas hydrophila*	Reduction in florfenicol MIC from 16 to 4 µg/mL and a FICI = 0.50. Synergistic antibiofilm activity at sub-MIC levels (rutin at 275 µg/mL and florfenicol at 4 µg/mL) with 82% biofilm reduction. Reduced the virulence by enhancing host immunity and genotoxic effects.	[142]
Florfenicol	Quercetin	*Aeromonas hydrophila*	Florfenicol MIC value was reduced 32 times (from 2.5 to 0.078 µg/mL) when combined with quercetin, FICI = 0.28 (synergy). Reduced bacterial viability by 60.5- and 115-fold, as well as the bacterial load in Cyprinus carpio tissues up to 610.6-fold, compared to the florfenicol group. Improved the survival rate of infected fish from 10% (control) to 90% (combined treatment).	[140]
Florfenicol	Luteolin	*Aeromonas hydrophila*	Luteolin exerted synergistic values (FICI = 0.125–0.375) and enhanced the survival rate and decreased the bacterial load of grass carp and ayu.	[143]
Florfenicol	Linalool	*Aeromonas hydrophila*	Synergistic antibacterial activity, inhibited biofilm formation, and haemolysis.	[144]
Tetracyclines				
Oxytetracycline	Tea polyphenols	*Vibrio parahaemolyticus*	The combination of 1/64 MIC (0.0024 mg/mL) of tea polyphenols and 1/8 MIC (1 µg/mL) of oxytetracycline resulted in a small synergistic FICI of 0.14. Improved the in vitro survival rate (53.3%) of *Exopalaemon carinicauda,* activities of digestive and immune enzymes, and increased the resistance against *V. parahaemolyticus.*	[145]
Tetracycline	*Litsea cubeba* EO	*Vibrio parahaemolyticus*	Synergistic effect (FICI = 0.31) in inhibiting growth and biofilm formation. Down-regulated the *tolC* and *ompW* involved in drug resistance.	[146]
Oxytetracycline	Linalool	*Aeromonas hydrophila*	Synergistic antibacterial activity, inhibited biofilm formation, and haemolysis.	[144]
	Citral	*Citrobacter freundii*		
Quinolones				
Enrofloxacin	Curcumin	*Aeromonas hydrophila*	Inhibited the growth, damaged the bacterial membrane, and increased K^+^ leakage.	[147]
Enrofloxacin	Barberine hydrochloride	*Edwardsiella ictaluri* (HSN-1)	MIC value of enrofloxacin was reduced from 0.025 to 0.003125 mg/mL (FICI = 0.625) when combined with a sub-MIC concentration of barberine.	[148]
β-lactams				
Carbenicillin	*Gracillaria* sp.	*Vibrio parahaemolyticus*	*Gracillaria* sp. potentiates the antibacterial activity of the antibiotic, the MIC of carbenicillin was reduced 4 times and showed a FICI = 0.26 (synergy). The bacterial growth was reduced to 4.1 log UFC/mL at 12 h of incubation, compared to 8.4 log UFC/mL of the antibiotic treatment.	[139]

## Data Availability

No new data were created or analyzed in this study. Data sharing is not applicable to this article.

## Abbreviations

The following abbreviations are used in this manuscript:

AHPND	Acute Hepatopancreatic Necrosis Disease
ARGs	Antibiotic Resistance Genes
BKD	Bacterial Kidney Disease
CFU	Colony Forming Units
DNA	Deoxyribonucleic Acid
EMS	Early Mortality Syndrome
EO	Essential Oil
EPS	Extracellular Polymeric Substances
FAO	Food and Agriculture Organization
FDA	Food and Drug Administration
FIC	Fractional Inhibitory Concentration
FICI	Fractional Inhibitory Concentration Index
GRAS	Generally Recognized As Safe
HGT	Horizontal Gene Transfer
MIC	Minimum Inhibitory Concentration
MBC	Minimum Bactericidal Concentration
MGEs	Mobile Genetic Elements
MRL	Maximum Residue Limit
QS	Quorum Sensing
ROS	Reactive Oxygen Species
VBNC	Viable But Non-Culturable
WHO	World Health Organization
